# Association Between Short-Form Video Use and Mental Health: Systematic Review and Meta-Analysis

**DOI:** 10.2196/82503

**Published:** 2026-03-18

**Authors:** Di Tang, Xin Zhang, Pengpeng Gou, Jie Feng, Rui Hu, Kim-wai Raymond Sum

**Affiliations:** 1 Department of Sports Science and Physical Education, Faculty of Education, Chinese University of Hong Kong, G09, Kwok Sports Building, Hong Kong, China (Hong Kong), 852 3943 6091; 2 The Nethersole School of Nursing, Faculty of Medicine, Chinese University of Hong Kong, Hong Kong, China (Hong Kong); 3 Faculty of Education, Central China Normal University, Wuhan, Hubei, China; 4 School of Sports Training, Wuhan Sports University, Wuhan, Hubei, China; 5 Center of Strength and Conditioning, Wuhan Sports University, Wuhan, Hubei, China; 6 Faculty of Sports Science, Ningbo University, Ningbo, Zhejiang, China; 7 School of Sports Science and Physical Education, Southwestern University of Finance and Economics, Chengdu, Sichuan, China

**Keywords:** short-form video, social media, mobile phone, addiction, mental health, meta-analysis

## Abstract

**Background:**

Short-form videos (SFVs) have emerged as a dominant trend in digital content sharing over the past decade, gaining rapid global popularity. An increasing number of studies have explored the association between SFV use and mental health, yet current empirical evidence remains inconsistent.

**Objective:**

This study aimed to provide a comprehensive synthesis examining the relationship between SFV usage and mental health outcomes, distinguishing between problematic and routine usage behaviors.

**Methods:**

Web of Science, PubMed, Scopus, Embase, PsycInfo, SportDiscus, and ProQuest were searched for relevant literature up to January 3, 2026. Statistical metrics reflecting the relationship between SFV use and mental health outcome indicators were extracted for meta-analysis, such as Pearson correlation coefficients, Spearman’s rank correlation coefficients, and *β* coefficients. For intervention and longitudinal studies, we extracted the baseline correlation coefficients. The Joanna Briggs Institute Critical Appraisal Checklist was used to assess the risk of bias.

**Results:**

A total of 58 studies, with a cumulative sample of 96,676 participants, were included in the analysis. Results showed no significant correlations between SFV use and positive psychological states, but significant positive correlations with negative psychological states, including depression (*r*=0.24, 95% CI 0.15-0.33), anxiety (*r*=0.26, 95% CI 0.17-0.35), stress (*r*=0.41, 95% CI 0.38-0.56), negative affect (*r*=0.22, 95% CI 0.10.33), loneliness (*r*=0.33, 95% CI 0.25-0.41), and boredom (*r*=0.42, 95% CI 0.29-0.53). Subgroup analyses revealed substantial differences between usage patterns: problematic use demonstrated significant negative associations with subjective well-being (*r*=−0.25, 95% CI −0.35 to −0.14) and positive correlations with adverse mental health outcomes, while routine usage showed no significant associations with negative affect and exhibited a negative but nonsignificant correlation with anxiety (*r*=−0.02, 95% CI −0.18 to 0.14). Additionally, while some studies reported significant associations between routine SFV use (time spent or frequency) and mental health outcomes, these findings were less consistent and showed smaller effect sizes compared to the more robust correlations found with problematic use measures. This suggests that the relationship between time spent on SFV and mental health may be less stable than that of problematic use patterns, highlighting the importance of considering qualitative aspects of usage rather than merely quantitative metrics.

**Conclusions:**

This meta-analysis suggests that SFV use is associated with adverse mental health outcomes. Future research should use objective measurement instruments to capture contemporary SFV usage patterns and differentiate between distinct types of engagement. Additionally, studies with greater geographical diversity and longitudinal or experimental designs are needed to establish causality and examine temporal changes in these associations.

## Introduction

In the past decade, short-form videos (SFVs) have emerged as a dominant trend in digital content sharing, rapidly gaining global popularity. Platforms such as TikTok (known as Douyin in China) have transformed the way users create and consume content by enabling the production and sharing of videos lasting only a few seconds to a few minutes [1]. By 2022, TikTok had surpassed 1.2 billion monthly active users, with the average user spending 168 minutes per day on the platform [2]. In China, approximately 95% of internet users engage in SFV consumption [3]. Historically, video-sharing platforms like YouTube primarily hosted long-form content [4]. However, in response to the growing popularity of SFVs, major platforms such as YouTube, Instagram, and Snapchat have integrated similar features to meet user demand [5]. SFVs have gained attention due to their ease of use and convenience, alongside their engaging nature, becoming a defining feature of modern social media. These platforms not only facilitate passive content consumption but also actively encourage user participation and creativity, reshaping self-expression and cultural exchange in the digital age [6,7].

Compared to other forms of social media, SFV apps have quickly captured significant market share by addressing users’ psychological needs, such as fostering social connections, providing emotional gratification, and fulfilling the desire for recognition [6]. One key factor driving this success is the availability of intuitive video editing tools, which empower users to easily become content creators, thereby enhancing engagement and participation [7]. For instance, Instagram reported that by 2020, more than 500 million accounts used its “Stories” feature daily, underscoring the growing appeal of SFV consumption [8]. The preference for SFVs can also be attributed to the fast-paced nature of modern life, where users prioritize convenience and brevity. Platforms offering SFV content are particularly appealing because they are user-friendly and rely on algorithm-driven personalization to deliver tailored content based on individual preferences [5]. This personalized approach not only improves user satisfaction but also boosts traffic, accelerating the widespread adoption of these platforms.

The diverse range of content available in SFVs further contributes to its widespread popularity. These videos span a variety of topics, including beauty, cooking, entertainment, education, health, and technology [5]. While traditional social media platforms often focus on lifestyle-oriented content, platforms like TikTok have differentiated themselves by emphasizing creative, unconventional, and often humorous videos. The majority of these videos are not professionally produced, which enhances their authenticity and relatability. TikTok, for example, primarily targets younger audiences with content such as music, dance, and viral comedic trends. In contrast, Douyin, its Chinese counterpart, caters to a broader demographic by featuring content that resonates with older users and focuses more heavily on everyday life [8]. This dual strategy has enabled TikTok to expand its user base and appeal to diverse cultural and demographic groups, cementing its role as a global leader in SFV content.

Despite the significant economic value brought by SFVs and their role in enriching modern entertainment, their rapid growth and potential for overuse have raised concerns among researchers about underlying health risks [6]. The convenience and entertainment value of SFVs make it easy for the public, especially younger users, to overuse them and struggle to control their viewing habits, resulting in increased time spent on these platforms. This trend exacerbates worries regarding negative health outcomes [6,9]. Current research indicates that excessive use of SFVs exhibits similarities to internet addiction or smartphone addiction, potentially leading to addictive behaviors and health issues, such as impairing social activities, interpersonal relationships, emotional stability, and psychological well-being [1,2,10,11]. Studies have shown that the algorithmic recommendation systems of SFV apps contribute to an information cocoon that increases users’ feelings of loneliness, which may subsequently lead to higher levels of depression [12]. Additionally, problematic use of SFVs has been associated with suicidal ideation and self-injurious behaviors [13]. Moreover, numerous studies highlight a positive correlation between SFV usage and negative emotions such as anxiety and stress, while revealing a negative correlation with well-being, self-esteem, and sleep quality [14-17]. However, the literature is far from conclusive. Some studies have reported no significant correlation between SFV usage and specific mental health indicators, such as depression and anxiety [17-19]. For example, Banjanin et al [20] found no significant correlation between depression and time spent on social media. Chung et al [21] and Zhai et al [22] found a positive relationship between SFVs and life satisfaction, and Chao et al [23] discovered that different usage patterns may have opposed effects on mental health. This body of research underscores the complexities surrounding the relationships in this domain [17].

In the current investigations examining the relationship between SFV usage and psychological health indicators, the selected metrics for assessing SFV usage can be broadly categorized into 2 types. The first type pertains to an individual’s general engagement with SFVs, encompassing several dimensions such as frequency, duration, and other indices reflecting the extent of usage [9,24-26]. While usage frequency quantifies the time an individual allocates to SFVs, it does not capture the psychological quality of that engagement. The second focuses on problematic usage, centering on addiction severity, dependency, and compulsive behavior [27-29]. Unlike simple usage intensity, problematic usage is characterized by psychological dependence, emotional distress, and functional impairment. Consequently, high-frequency use may simply reflect strong interest or leisure engagement without necessarily implying maladaptive outcomes. Therefore, treating these 2 types of metrics as interchangeable is methodologically problematic. Specifically, the association between usage behavior and psychological health may vary significantly depending on whether usage is operationalized as addiction or problematic dependency versus simply as daily time spent or frequency. For instance, research has reported that active usage of TikTok is significantly and negatively correlated with anxiety [30], whereas problematic usage of SFVs was reported to have a significant positive correlation with anxiety in another study [31]. This distinction is likely a primary driver of the inconsistencies observed in the existing literature. Furthermore, variations in research quality and design (such as sample size, outcome selection, and measurement methodologies) may further obscure the true relationship between SFV usage and mental health.

Despite the growing body of literature on the relationship between SFV usage and health, there remains a lack of systematic synthesis of existing findings. Given the inconsistencies in prior findings, this study aims to clarify the relationship between SFV use and mental health through a systematic review and meta-analysis. Additionally, by distinguishing between different usage patterns, this research seeks to investigate potential heterogeneity in the impact of SFV usage on mental health outcomes.

## Methods

The systematic review adhered to the 2020 guidelines established by the PRISMA (Preferred Reporting Items for Systematic Reviews and Meta-Analyses) [32] (Checklist 1). In addition, the review protocol was registered in advance in the International Prospective Register of Systematic Reviews database (Registration ID: CRD420251015620).

### Search Strategy

Web of Science, PubMed, Scopus, Embase, PsycInfo, SportDiscus, and ProQuest were searched to identify relevant literature from the inception of each database to January 3, 2026. The search terms combined variations of and specific health-related outcomes, such as depression, well-being, and anxiety. A detailed description of the search strategy can be found in the Multimedia Appendix 1. All references retrieved from the searches were organized using EndNote 20 (Clarivate) software.

### Eligibility Criteria

The selection criteria imposed no restrictions on participants’ sex or age. Regarding study design, we primarily included cross-sectional studies. However, longitudinal studies or interventional trials were also deemed eligible if they performed a cross-sectional analysis of baseline data—specifically, analyzing the relationship between SFV use and mental health outcomes before the commencement of any intervention or follow-up period—and reported the relevant correlation coefficients.

We operationally defined SFVs as video content lasting from several seconds to a few minutes [33,34], without imposing restrictions on specific platforms or content categories. However, for multifunctional video platforms not exclusively dedicated to SFVs (eg, Instagram), inclusion was restricted to studies that specifically isolated usage behaviors related to the SFV feature (eg, intensity of use or addiction to Instagram Reels), rather than general platform usage. Metrics for SFV use were categorized into regular usage indicators, including frequency, daily and weekly duration, as well as problematic use, which encompassed measures of video addiction, dependency levels, and indicators of excessive use. Regarding mental health, studies were required to assess mental health outcomes quantitatively. These constructs could include subjective measures (eg, self-reported or perceived health) or objective measures (eg, standardized tests). To ensure systematic categorization, identified health constructs were classified into mental health domains based on the World Health Organization’s International Classification of Diseases [35].

Crucially, this study focuses exclusively on users’ autonomous SFV usage behaviors. Therefore, specific interventional experiments involving forced exposure to predetermined content or artificial viewing tasks were excluded. Instead, included studies operationalized autonomous usage primarily through self-reported measures of usage frequency, duration, or validated intensity scales. Furthermore, conference abstracts lacking full texts or complete data, review studies, qualitative research, editorials, and letters were excluded from consideration. Non-English or non-Chinese literature was also excluded.

### Study Selection

After removing duplicates, 2 reviewers (DT and XZ) independently screened the titles and abstracts. Records that did not align with the research objectives, such as those failing to report mental health outcomes, not focusing on SFV users, or having ineligible publication types or study designs, were excluded. Subsequently, the full texts of the remaining articles were retrieved and assessed independently by the 2 reviewers to determine final inclusion based on the criteria. During the literature screening process, any disagreements between the 2 reviewers were resolved through discussion to reach a consensus. In cases where consensus could not be achieved, a third reviewer (RH) was available to make a final determination. To ensure the consistency of the screening process, we calculated the interrater reliability using Cohen kappa coefficient, which indicated strong agreement between the reviewers (*κ*=0.83).

### Risk of Bias

Two reviewers (DT and XZ) independently evaluated the quality of the included studies using the Joanna Briggs Institute (JBI) critical appraisal checklist for cross-sectional studies. Disagreements were resolved by discussion or, if necessary, consultation with a third reviewer (RH). The assessment covered three key domains: (1) sample and setting (inclusion criteria and subject description); (2) measurement validity (exposure, condition, and outcome measurement); and (3) confounding and analysis (identification or management of confounders and statistical appropriateness). Although some included studies used a longitudinal or interventional design, our analysis specifically concentrated on baseline data collected before any intervention. Therefore, we restricted our quality assessment to the cross-sectional component of these studies to evaluate the validity and reliability of the baseline data sources, confirming the appropriateness of the JBI cross-sectional checklist for this evaluation.

### Data Extraction and Analysis

The extracted data included study design, sample size, participants’ age, country, SFV platforms, and measured mental health outcomes. Additionally, statistical metrics reflecting the relationship between SFV use and mental health outcome indicators were extracted, such as Pearson correlation coefficients, Spearman’s rank correlation coefficients, and *β* coefficients. For intervention and longitudinal studies, we extracted the correlation coefficients at baseline.

Data analysis was carried out using Review Manager version 5.3 (The Cochrane Collaboration) and R version 4.4.2 (R Core Team). To achieve an approximate normal distribution for data synthesis, correlation coefficients (*r*) were converted to Fisher *Z*-scores. In instances where correlation coefficients were not reported directly, we used formulas recommended by the Cochrane Handbook for Systematic Reviews to transform other measures of effect size into correlation coefficients [36]. Quantitative synthesis was performed only when the relationship between a specific mental health outcome and SFVs was reported by more than 3 studies. Otherwise, a narrative discussion was conducted for the outcomes.

Statistical heterogeneity was quantified using the *I*^2^ statistic and the Q test. Random-effects models were used when the heterogeneity of the studies was high (*I*^2^>50%), while fixed-effects models were applied when the heterogeneity was low (*I*^2^≤50%).

Based on the existing research, we conducted further subgroup analyses of SFV use by categorizing usage indicators into two types: problematic use, which included measurements of SFV addiction, dependence, and overuse; and routine use, which included daily use duration or frequency. We further explored the relationship between these 2 types of usage indicators and mental health to determine whether there were differences in how these indicators were associated with mental health outcomes.

Potential publication bias was assessed through visual inspection of funnel plots, where asymmetry was evaluated. In accordance with the recommendations of the Cochrane Handbook, for outcomes including 10 or more studies, we further conducted quantitative assessments using Egger’s linear regression test and Begg’s rank correlation test. A *P* value of less than .05 was considered to indicate statistically significant publication bias.

Sensitivity analyses were performed to evaluate the robustness of the pooled results. First, we conducted a “leave-one-out” analysis to determine if any single study disproportionately influenced the overall effect size. Second, we assessed the impact of study quality on the outcomes by excluding studies classified as low quality based on the critical appraisal checklist.

## Results

### Search Results

**Figure 1.**
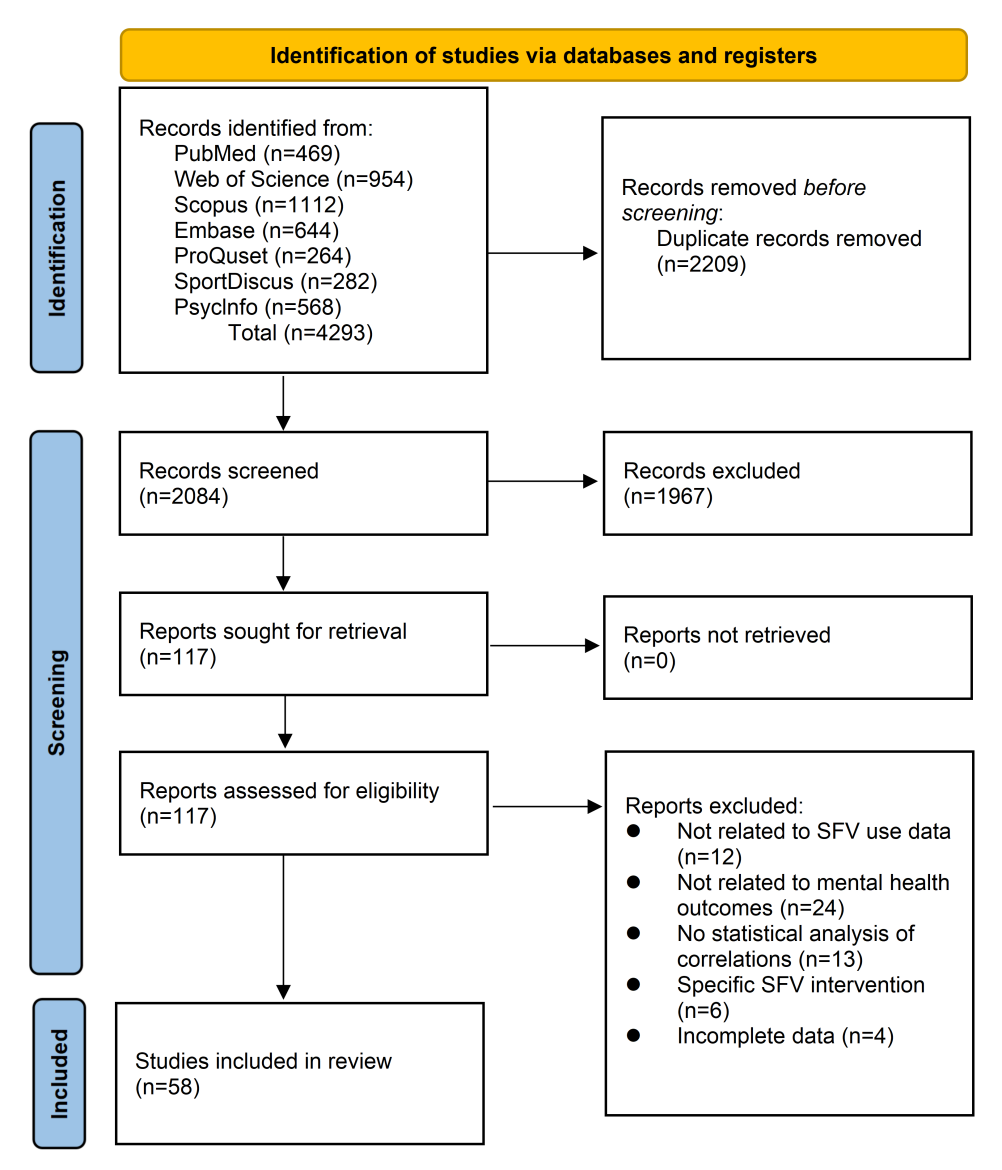
PRISMA (Preferred Reporting Items for Systematic Reviews and Meta-Analyses) flow chart of the identification process. SFV: short-form video.

A total of 4293 articles were retrieved from the databases. Figure 1 illustrates the identification process. Ultimately, 58 articles were included in the review, of which 53 articles were incorporated into the meta-analysis. Figure 1 presents the identification process using the PRISMA flow diagram.

### Characteristics of Studies

Among the 58 included studies, the majority were conducted in China (n=55), while a smaller number originated from other countries, including the United Arab Emirates (n=1), Pakistan (n=1), and the United States (n=1). The total accumulated sample size across all studies was 96,676, with individual study sample sizes ranging from 54 to 16,765. The mean age of participants ranged from 11.69 (SD 1.11) to 70.65 (SD 6.40) years, with some studies targeting specific populations, such as middle school students [37] and college students [29,38], while others included broader age ranges, such as 18‐95 years [26]. Only 16 articles reported the specific SFV platforms used. The primary platforms identified were TikTok, YouTube (YouTube Shorts), and Instagram (Instagram Reels). All studies were published between 2021 and 2025.

In terms of study design, 52 studies used a cross-sectional approach, 4 used a prospective design, 1 was an intervention study, and 1 adopted a mixed methods framework. SFV use was analyzed in various forms across the studies. SFV addiction was the most frequently examined behavior, reported in 52 studies, followed by daily use in 15 studies, problematic use in 5 studies, and overuse or dependence in a smaller subset [38-40]. Primary mental health outcomes assessed in these studies included depression (n=19), anxiety (n=13), loneliness (n=7), and well-being (n=12). Additional reported outcomes included stress, boredom, affect, suicidal ideation, and emotional dysregulation. Detailed study characteristics are shown in Multimedia Appendix 2.

### Risk of Bias

Quality assessment of the 58 included studies yielded an overall mean quality score of 79.1% (SD 14.3%). Among the included studies, 27 (46.6%) achieved high quality scores of greater than or equal to 87.5%. The quality assessment revealed significant variability across methodological domains. The highest compliance rates were observed for item 2 (detailed description of study subjects and setting; 96.6%), item 7 (valid and reliable outcome measurement; 96.6%), and item 8 (appropriate statistical analysis; 94.8%). Conversely, substantial methodological limitations were identified in item 1 (clear definition of inclusion criteria; 60.3%). Detailed quality assessment results were provided in Multimedia Appendix 3.

### Meta-Analysis

A total of 53 studies were included in the meta-analysis, which examined psychological outcomes categorized into negative psychological states (including depression, stress, anxiety, loneliness, boredom, and negative affect) and positive psychological states (including subjective well-being and positive affect). Studies that were not included in the meta-analysis were synthesized narratively.

### SFV Use and Positive Psychological States

**Figure 2.**
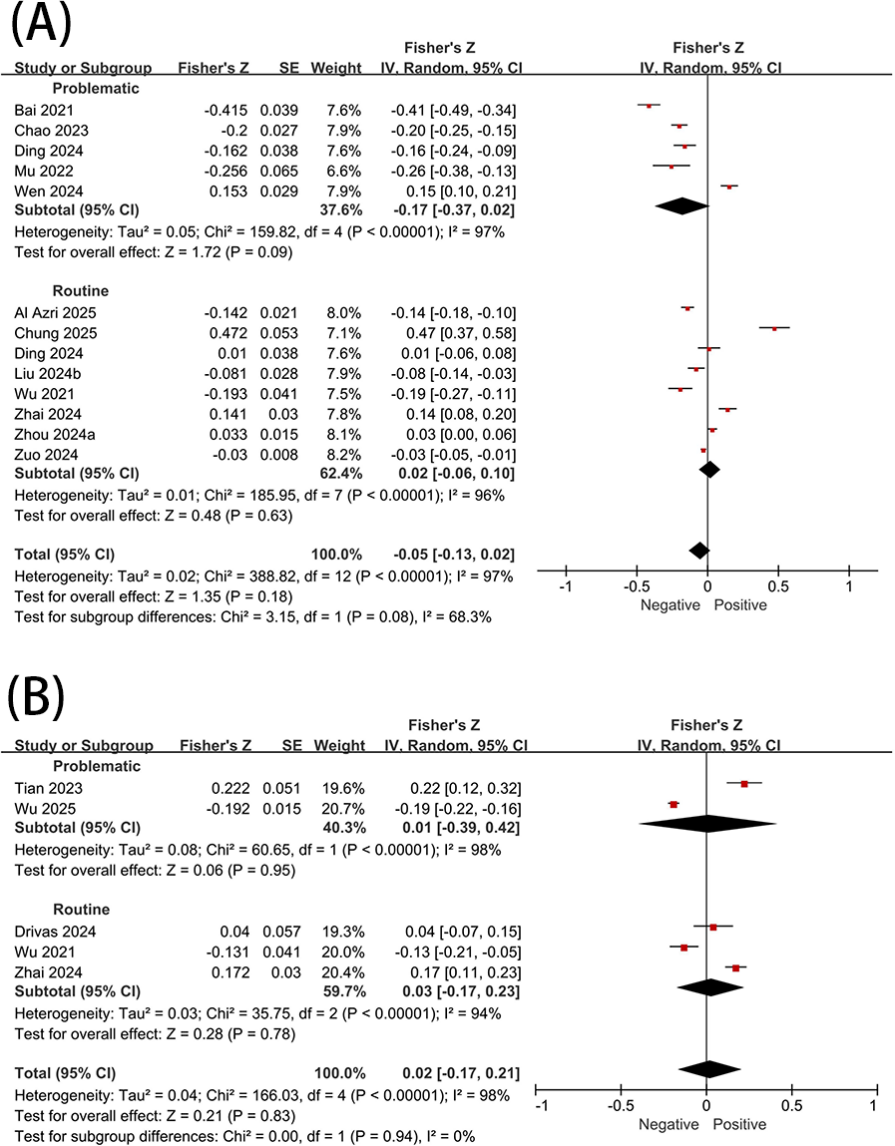
Association between short-form video use and (A) well-being and (B) positive affect [21-23,26,27,34,40-48].

As shown in Figure 2, no significant correlations were observed between SFV use and either subjective well-being or positive affect. However, the 2 subgroups, problematic and routine use, exhibited marginally significant heterogeneity in their associations with subjective well-being (*P*=.08; *I*^2^=68.3%). After excluding the study by Wen [40], problematic SFV use demonstrated a significant negative correlation with subjective well-being (*r*=−0.25, 95% CI −0.35 to −0.14).

### SFV Use and Negative Psychological States

**Figure 3.**
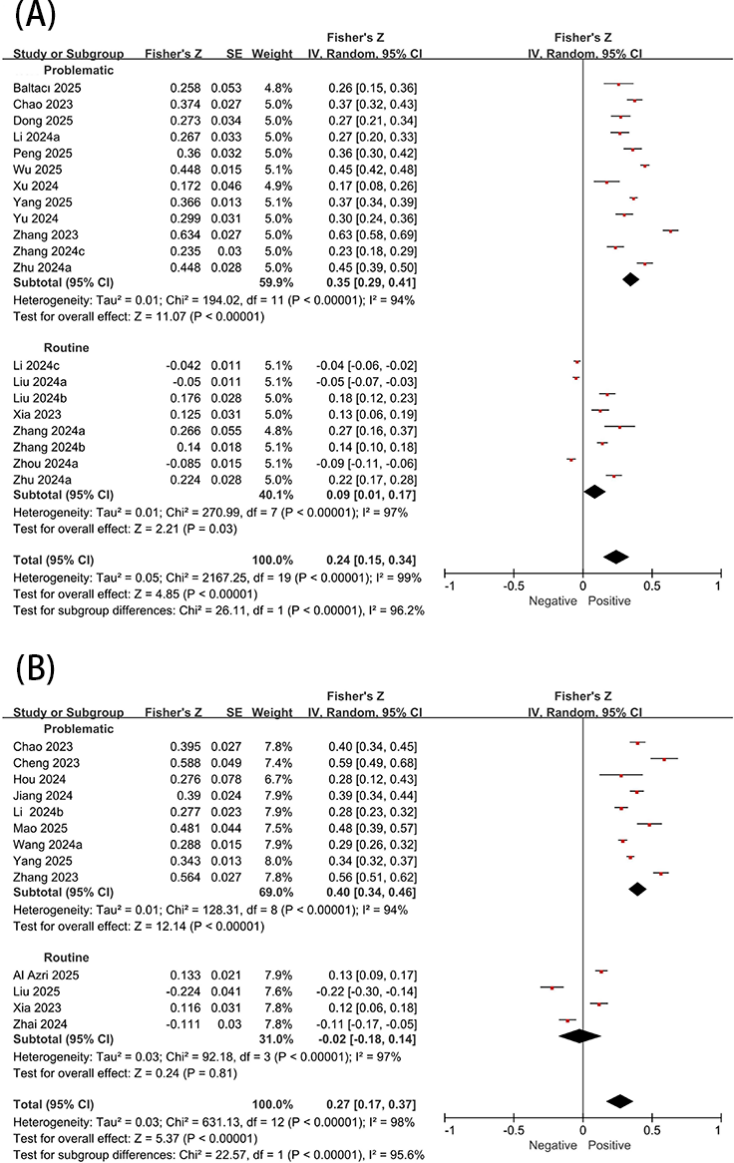
Association between short-form video use and depression (A) and anxiety (B) [13,22,23,25,28,29,31,38,39,43-45,47,49-62].

As shown in Figure 3A, SFV use demonstrated a small but significant positive correlation with depression (*r*=0.24, 95% CI 0.15-0.33). Subgroup analysis revealed significant heterogeneity between the problematic and routine group (*P*<.001; *I*^2^=96.2%). Problematic use demonstrated a significant moderate positive correlation with depression (*r*=0.34, 95% CI 0.28-0.39), while routine use showed a weak but significant positive association with depression (*r*=0.09, 95% CI 0.01-0.17). However, in the leave-one-out sensitivity analysis, the association between general use and depression became statistically nonsignificant when [25,25], Zhang 2024 [49], Xia 2024 [50], or [44] were excluded individually. This suggests that the current evidence regarding the positive correlation between daily use and depression is not robust, and these findings should be interpreted with caution.

SFV use was significantly positively correlated with anxiety (*r*=0.26, 95% CI 0.17-0.35). Significant heterogeneity was found between the subgroups of problematic and routine use (*P*<.001; *I*^2^=95.6%). Problematic use showed a moderate to large positive correlation with anxiety (*r*=0.38, 95% CI 0.33-0.43), while routine use demonstrated a nonsignificant negative association with anxiety (Figure 3B).

**Figure 4.**
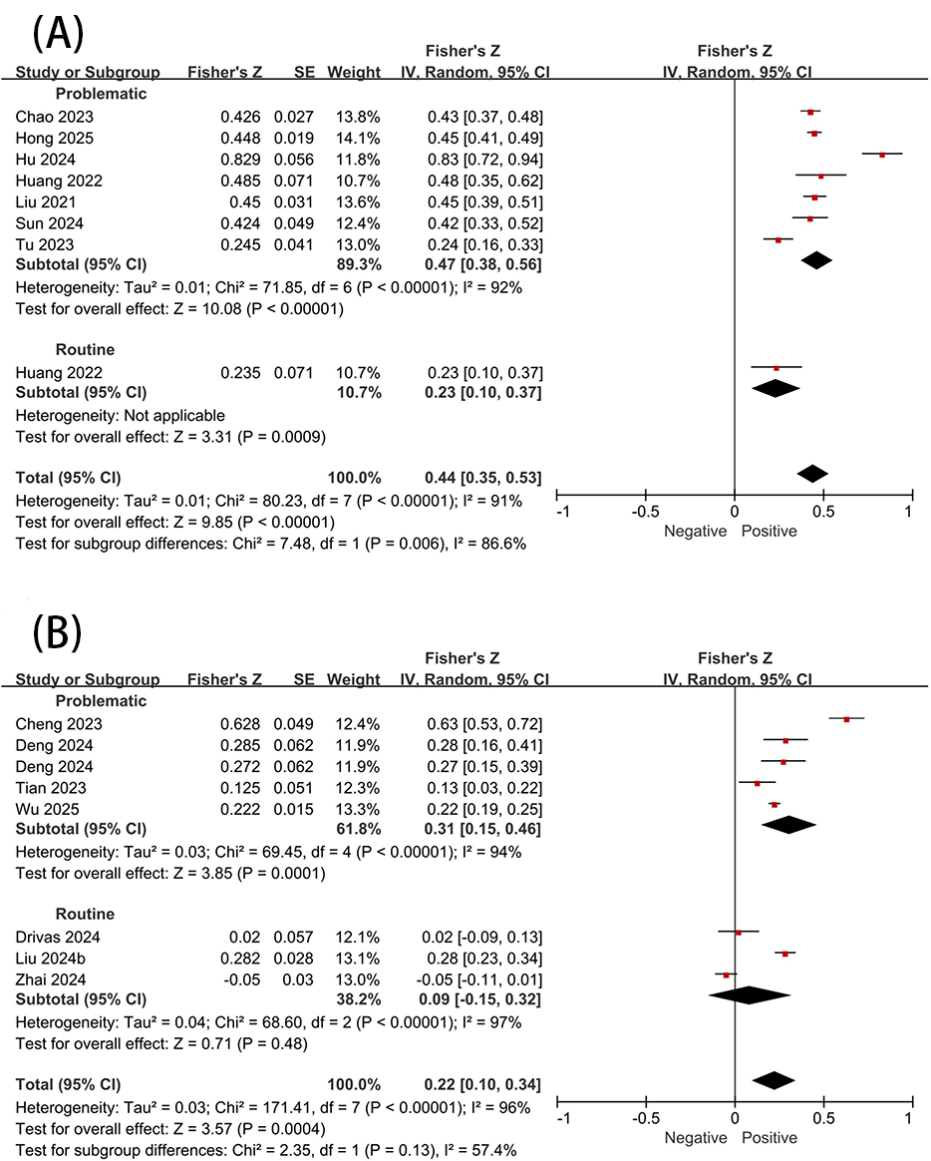
Association between short-form video use and (A) stress and (B) negative affect [3,14,15,22,23,47,48,57,58,63-66].

SFV use was found to be significantly associated with stress (*r*=0.41, 95% CI 0.38-0.56). Significant heterogeneity was also found between the subgroups of problematic and routine use (*P*=.006; *I*^2^=86.6%). Specifically, problematic use demonstrated a moderate to large positive correlation with stress (*r*=0.44, 95% CI 0.36-0.51), whereas routine use showed a small to moderate positive association with stress (*r*=0.23, 95% CI 0.1-0.35). However, this result should be interpreted with caution as the routine use subgroup comprised only 1 study (Figure 4A). Regarding negative affect, SFV use was also found to be significantly positively associated (*r*=0.22, 95% CI 0.1-0.33). Problematic use exhibited a moderate and significant positive correlation with negative affect (*r*=0.3, 95% CI 0.150.43), while no significant association was observed between routine and negative affect (Figure 4B).

**Figure 5.**
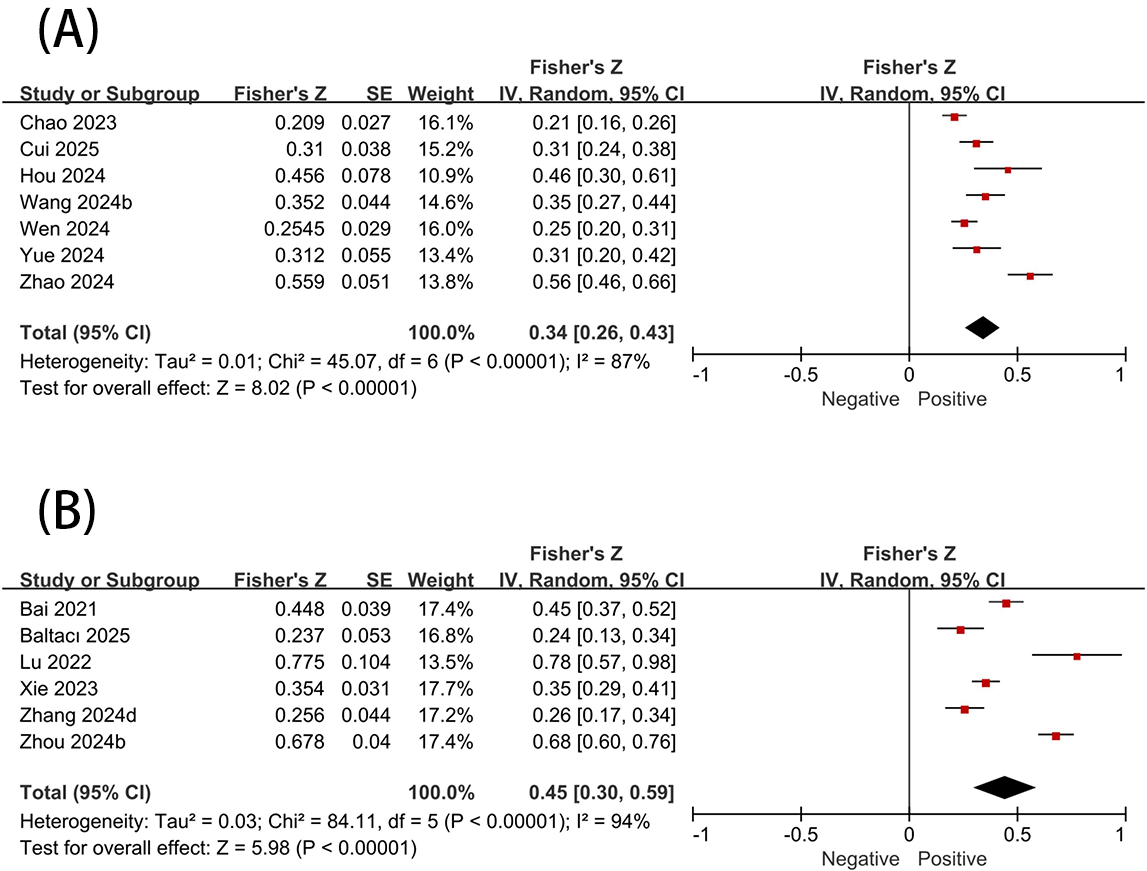
Association between short-form video use and (A) loneliness and (B) boredom [23,27,40,51,59,69,72-78].

For SFV use and its associations with loneliness and boredom, only studies examining the relationships between problematic use and these 2 variables were sufficient for analysis. Therefore, no further subgroup analyses were conducted. Problematic SFV use demonstrated significant positive correlations with both loneliness (*r*=0.33, 95% CI 0.25-0.41) and boredom (*r*=0.42, 95% CI 0.29-0.53) (Figure 5).

Additionally, in studies not included in the meta-analysis, we found that problematic SFV use was positively associated with suicidal attempts (*r*=0.07; *P*<.05) [13]. SFV addiction showed positive associations with emotional suppression (*r*=0.29; *P*<.001) [37], alexithymia (*r*=0.339; *P*<.001) [67], emptiness (*r*=0.66, *P*<.001) [68], and neuroticism (*r*=0.3; *P*<.01) [69], (*r*=0.27; *P*<.01) [70], and (*r*=0.39; *P*<.001) [28]. And SFV addiction was also found to be significantly associated with adolescent emotional dysregulation [71].

### Publication Bias and Sensitivity Analysis

Funnel plots for all analyses showed no obvious asymmetry (Multimedia Appendix 4). Furthermore, for outcomes including 10 or more studies, Egger and Begg tests consistently yielded *P* values greater than .05, indicating no significant evidence of publication bias. Additionally, only 1 study was classified as low quality (JBI score <50%) [79]. Although this study was identified as low quality, it was primarily excluded from the final meta-analysis because insufficient studies reported the specific mental health indicators it analyzed to allow for pooling. Instead, it was synthesized narratively. Consequently, a sensitivity analysis comparing results before and after excluding low-quality studies was not feasible. Leave-one-out sensitivity analyses showed that the statistical significance of our main results remained consistent, confirming their overall robustness. However, in the subgroups examining the relationship between depression and routine use, and well-being and problematic use, excluding specific studies altered the significance of the results, as detailed in the Results section. Consequently, the findings concerning these specific associations appear unstable and warrant cautious interpretation.

## Discussion

### Principal Findings

This systematic review with meta-analysis provides a comprehensive synthesis of the relationship between SFV use and mental health. It is the first to distinguish between problematic and routine usage behaviors, revealing their divergent associations with different mental health outcomes. Analyses of current evidence indicated that SFV use demonstrated no significant associations with indicators of positive mental health, including subjective well-being and positive affect. Conversely, significant associations emerged between SFV use and various negative mental health states, including depression, anxiety, stress, negative affect, loneliness, and boredom.

Notably, our subgroup analyses revealed substantial differences between problematic and routine usage. Specifically, problematic SFV use demonstrated a significant negative association with subjective well-being and positive correlations with adverse mental health outcomes. In contrast, routine usage behaviors showed no significant associations with negative affect, and surprisingly, exhibited a significant negative association with anxiety, suggesting that nonproblematic routine engagement with SFV platforms may even serve an anxiety-relieving function for some users [22,30]. In addition, current evidence on the relationship between routine use and depression or stress remains inconclusive. The pooled correlations between routine SFV use and depression were sensitive to the removal of individual studies, with statistical significance changing in several leave-one-out analyses, suggesting that these findings lack robustness. These findings also align with established literature indicating that heightened usage duration and elevated dependency levels are associated with increased vulnerability to adverse mental health outcomes and diminished positive psychological functioning compared to moderate or low-frequency usage patterns [23,44]. The marked distinction between problematic and routine usage in relation to mental health outcomes underscores the importance of considering qualitative differences in how individuals engage with SFV platforms, rather than treating SFV use as a monolithic construct. Overall, these findings support a more nuanced conceptualization of SFV use and its relationship with mental health, emphasizing that both the nature and extent of engagement are critical determinants of psychological outcomes.

Overall, in analyzing the relationship between problematic SFV use and mental health, the results of this meta-analysis broadly align with previous findings on problematic internet, smartphone, or social media use, both demonstrating correlations with decreased mental health and various mental health issues [80-83]. Prior research has also identified significant positive correlations between screen time and internalizing and externalizing behavior problems [84]. This suggests that despite differences in content and usage patterns between SFVs and traditional social media or video platforms, problematic SFV use may share similar mechanisms of impact on mental health with problematic internet, smartphone, and social media use.

However, it is noteworthy that our study found the strength of the association between problematic SFV use and negative mental health outcomes to be higher than previously established correlations for problematic phone use, internet use, social media use, and screen time [80-82,85,86]. In this meta-analysis, problematic SFV use shows moderate correlations with depression, anxiety, stress, loneliness, and boredom. This numerical difference might support that SFVs constitute a distinct media format, emphasizing the necessity for specialized research focused on SFVs rather than simply categorizing them under general “screen time” or “social media use” when considering their relationship with mental health.

First, the algorithmic recommendation mechanisms of SFV platforms may more intensely reinforce user immersion and platform stickiness [87-89]. Unlike traditional media, SFV platforms continuously deliver highly personalized content aligned with users’ interests through sophisticated algorithms, creating reinforcement loops that potentially lead to information cocoons and cognitive biases [90], which may further exacerbate negative mental health outcomes [12,91].

Furthermore, the short duration and rapid switching nature of SFVs may significantly impact users’ attention regulation and cognitive processing abilities. Long-term exposure potentially leads to attention fragmentation and increased cognitive burden, affecting daily functioning [78,92]. The highly condensed nature of SFV content intensifies information input, worsening cognitive load and creating information overload that may harm mental health [40].

Additionally, the rise of SFV platforms has transformed user roles, with increasing numbers of users shifting from passive information recipients to content creators compared to traditional social media or mobile phone usage [7,93]. This transition requires users to spend more time creating content on the platform, fundamentally changing usage patterns. Particularly on platforms like TikTok, users can not only create content but also monetize their videos, establishing content creation as a career option [94,95]. This behavioral pattern transformation potentially introduces psychological impacts distinctly different from those associated with traditional video consumption behaviors. For long-term content creators on SFV platforms, metrics such as view counts and video revenue may produce additional psychological effects that differ significantly from the psychological experiences of regular users who primarily engage in viewing activities [95-98]. However, current research has not effectively differentiated between these behavioral patterns, instead using simple measures of usage frequency and dependence to quantify SFV engagement without a nuanced distinction of qualitatively different usage patterns. This limitation may partly explain the substantial heterogeneity observed in our meta-analysis. Previous research on social media and internet usage has established that different usage patterns may exhibit entirely distinct relationships with mental health outcomes, such as differences between active versus passive engagement styles [22,30,86]. Future research needs to explore not only variations in usage intensity but also examine different usage patterns and their differential impacts on mental health.

Furthermore, the discussion regarding causality warrants further elaboration in the future. Although existing evidence reveals significant associations between SFV use and mental health, the cross-sectional nature of the included studies limits our ability to determine the direction of causality. This relationship is likely complex and multidimensional. First, psychological states may serve as antecedents to SFV use. For instance, individuals may engage with SFVs as a coping mechanism or for emotional regulation in response to boredom, loneliness, or stress [99,100]. Second, SFV use may precede psychological states. For example, problematic use may lead to sleep displacement or increased social comparison, thereby precipitating stress or anxiety [44,101]. Finally, a reciprocal relationship is plausible, analogous to the “internet paradox” [102] or the “reinforcing spirals model” [103]. In this scenario, adverse psychological states may precipitate problematic use, which subsequently aggravates the initial mental health concerns. Future longitudinal inquiries are indispensable for elucidating these temporal dynamics.

The effect sizes for correlations between both routine usage and problematic usage with mental health outcomes exhibited substantial heterogeneity in our findings. This variability likely stems from the wide age ranges, demographic characteristics, usage patterns, and intensity levels represented across existing studies. This heterogeneity suggests that the impact of SFV use on mental health may be influenced by multiple moderating factors, consistent with previous meta-analytic findings for other digital media. Future research should prioritize more specific investigations to analyze particular effect sizes for specific usage patterns and demographic groups. The differences in measurement instruments highlight the importance of developing and implementing standardized, validated assessment tools for SFV usage patterns and addiction. Specifically, researchers should consider establishing measures that distinguish between content creation and content consumption to more accurately reflect the usage dynamics of modern SFV platforms.

### Limitations and Future Directions

Several limitations of the current meta-analysis warrant consideration. First, the included studies were predominantly conducted in China, with limited data from other countries and regions. Given that research has demonstrated the importance of considering cultural and educational backgrounds when investigating problematic smartphone use, caution should be exercised when generalizing these findings to populations in different countries. Previous research on social media has revealed significant variations in associations across different countries and regions [81,85], highlighting the need for future cross-cultural investigations of SFV use.

Second, the limited number of available studies precluded detailed subgroup analyses, assessments of publication bias, and examination of potential moderators such as age, gender, and educational level. Understanding these factors would provide valuable insights into how SFV use and mental health relationships vary across demographic profiles. Future studies should therefore prioritize comprehensive reporting of both participant characteristics (age, gender, and education) and SFV usage patterns (platforms and content types). Particularly important is the distinction between content creators and passive viewers, alongside demographic information. Such detailed data are essential for developing a more nuanced understanding of these relationships.

Third, most of the included studies relied on self-report measures with considerable heterogeneity in assessment instruments, and many studies lacked validated methods or objective standard measurements for SFV use, such as actual usage time and frequency data obtained directly from platform analytics. To enhance cross-study comparability, there is an urgent need to develop and validate standardized measurement tools specifically designed for SFV use or addiction. Future research should differentiate between various SFV usage patterns and explore the potential differential associations between qualitatively distinct forms of engagement and mental health outcomes. Additionally, researchers should consider using more objective approaches to assess users’ mental health status, including a diversified indicator system comprising physiological measures, biofeedback techniques, and behavioral data.

Finally, most of the included studies employed cross-sectional designs, which preclude causal inferences and directional conclusions regarding the relationships observed. Future research should prioritize longitudinal investigations to further elucidate causal relationships and examine whether these associations change over time.

### Conclusion

This meta-analysis reveals that problematic SFV use is significantly associated with adverse mental health outcomes and reduced psychological well-being. Our findings highlight the distinct relationships between problematic versus routine usage patterns and mental health. Future research should develop standardized assessment tools that better capture contemporary SFV usage patterns and differentiate between varied usage behaviors. Longitudinal and experimental studies are needed to clarify causal relationships and examine whether these associations change over time. Additionally, as current evidence is predominantly based on Chinese samples, there is a critical need for data from diverse geographic regions to understand how these relationships may vary across different cultural contexts and user populations worldwide.

## Supplementary material

10.2196/82503 Multimedia Appendix 1 Search strategy.

10.2196/82503 Multimedia Appendix 2 Characteristics of the included studies.

10.2196/82503 Multimedia Appendix 3 Quality assessment of included studies.

10.2196/82503 Multimedia Appendix 4 Publication bias.

10.2196/82503 Checklist 1 PRISMA checklist.

## Abbreviations

JBI: Joanna Briggs Institute
PRISMA: Preferred Reporting Items for Systematic Reviews and Meta-Analyses
SFV: short-form video
