# Supplementary material for: Association Between Short-Form Video Use and Mental Health: Systematic Review and Meta-Analysis
Source: J Med Internet Res. 2026 Mar 18;28:e82503. doi: 10.2196/82503 (PMC12998613; doi:10.2196/82503)
Supplement: Multimedia Appendix 1 [file jmir-v28-e82503-s001.docx]

**Supplemental 1 Search strategy**

**Search terms:**

Exposure: “short video*” OR “short-form video*” OR “short form video*” OR “Tik Tok” OR Douyin OR “Instagram Reels” OR “YouTube Shorts” OR “SnackVideo”

Outcomes: “mental health” OR “psychological health” OR depress* OR anxiety OR stress OR “well-being” OR “self-esteem” OR distress OR loneliness OR emotion*

**PUBMED 469**

#1 ("short video*"[Title/Abstract] OR "short-form video*"[Title/Abstract] OR "short form video*"[Title/Abstract] OR "tik tok"[Title/Abstract] OR Douyin[Title/Abstract] OR "Instagram Reels"[Title/Abstract] OR "YouTube Shorts"[Title/Abstract] OR "SnackVideo"[Title/Abstract]) AND ("mental health"[Title/Abstract] OR "psychological health"[Title/Abstract] OR depress*[Title/Abstract] OR anxiety[Title/Abstract] OR stress[Title/Abstract] OR "well-being"[Title/Abstract] OR "self-esteem"[Title/Abstract] OR distress[Title/Abstract] OR loneliness[Title/Abstract] OR emotion*[Title/Abstract]) Filters: Chinese, English

#2 ("short video*" OR "short-form video*" OR "short form video*" OR "tik tok" OR Douyin OR "Instagram Reels" OR "YouTube Shorts" OR "SnackVideo"[MeSH Terms]) AND ("mental health" OR "psychological health" OR depress* OR anxiety OR stress OR "well-being" OR "self-esteem" OR distress OR loneliness OR emotion*[MeSH Terms]) Filters: Chinese, English

#1 OR #2

**Scopus 1112**

( TITLE-ABS-KEY ( "short video*" OR "short-form video*" OR "short form video*" OR "tik tok" OR douyin OR "Instagram Reels" OR "YouTube Shorts" OR "SnackVideo" ) AND TITLE-ABS-KEY ( "mental health" OR "psychological health" OR depress* OR anxiety OR stress OR well-being OR self-esteem OR distress OR loneliness OR emotion* ) ) AND ( LIMIT-TO ( LANGUAGE , "English" ) OR LIMIT-TO ( LANGUAGE , "Chinese" ) )

**Web of Science 954**

(TS=(“short video*” OR “short-form video*” OR “short form video*” OR “tik tok” OR Douyin OR “Instagram Reels” OR “YouTube Shorts” OR “SnackVideo”)) AND TS=(“mental health” OR “psychological health” OR depress* OR anxiety OR stress OR “well-being” OR “self-esteem” OR distress OR loneliness OR emotion* )

**Embase 644**#1 ("short video*"[Title/Abstract] OR "short-form video*"[Title/Abstract] OR "short form video*"[Title/Abstract] OR "tik tok"[Title/Abstract] OR Douyin[Title/Abstract] OR "Instagram Reels"[Title/Abstract] OR "YouTube Shorts"[Title/Abstract] OR "SnackVideo"[Title/Abstract]) AND ("mental health"[Title/Abstract] OR "psychological health"[Title/Abstract] OR depress*[Title/Abstract] OR anxiety[Title/Abstract] OR stress[Title/Abstract] OR "well-being"[Title/Abstract] OR "self-esteem"[Title/Abstract] OR distress[Title/Abstract] OR loneliness[Title/Abstract] OR emotion*[Title/Abstract])

Filters: Chinese, English

#2 ("short video*" OR "short-form video*" OR "short form video*" OR "tik tok" OR Douyin OR "Instagram Reels" OR "YouTube Shorts" OR "SnackVideo"[Emtree Terms]) AND ("mental health" OR "psychological health" OR depress* OR anxiety OR stress OR "well-being" OR "self-esteem" OR distress OR loneliness OR emotion*[Emtree Terms])

Filters: Chinese, English

#1 OR #2

**ProQuset 264**

(TI, AB, SU("short video*" OR "short-form video*" OR "short form video*" OR "TikTok" OR Douyin OR "Instagram Reels" OR "YouTube Shorts" OR "SnackVideo") ) AND (TI, AB, SU("mental health" OR "psychological health" OR depress* OR anxiety OR stress OR "well-being" OR "self-esteem" OR distress OR loneliness OR emotion*) )

**SportDiscus 282**

("short video*" OR "short-form video*" OR "short form video*" OR "TikTok" OR "Douyin" OR "Instagram Reels" OR "YouTube Shorts" OR "SnackVideo") AND ("mental health" OR "psychological health" OR depress* OR anxiety OR stress OR "well-being" OR "self-esteem" OR distress OR loneliness OR emotion*)

**Psyclnfo 568**

("short video*" OR "short-form video*" OR "short form video*" OR "TikTok" OR "Douyin" OR "Instagram Reels" OR "YouTube Shorts" OR "SnackVideo") AND ("mental health" OR "psychological health" OR depress* OR anxiety OR stress OR "well-being" OR "self-esteem" OR distress OR loneliness OR emotion*)
