# Supplementary material for: Association Between Short-Form Video Use and Mental Health: Systematic Review and Meta-Analysis
Source: J Med Internet Res. 2026 Mar 18;28:e82503. doi: 10.2196/82503 (PMC12998613; doi:10.2196/82503)
Supplement: Multimedia Appendix 2 [file jmir-v28-e82503-s002.docx]

| **Supplemental 2 Characteristics of the included studies and quality assessment** | | | | | | | |
| --- | --- | --- | --- | --- | --- | --- | --- |
| **Study** | **Design** | **Country** | **Sample (n, male)** | **Age (SD)** | **SFV use** | **Platform** | **Mental health outcomes** |
| Abulibdeh 2024 [1] | Cross-sectional | UAE | 54 (11) | NR | SFV addiction | Instagram, YouTube | Overall mental health |
| Al Azri 2025 [2] | Cross-sectional | Oman | 2285 (537) | 22.6 (4.14) | Daily use | Instagram | Anxiety, Well-being |
| Asad 2022 [3] | Cross-sectional | Pakistan | 350 (NR) | 18—30 | SFV addiction | TikTok | Neuroticism |
| Bai 2021 [4] | Cross-sectional | China | 656 (237) | NR | Problematic use | NR | Well-being, Boredom proneness |
| Baltacı 2025 [5] | Cross-sectional | Turkey | 361 (136) | 16.11 (1.41) | SFV addiction | NR | Depression, Boredom |
| Chao 2023 [6] | Cross-sectional | China | 1346 (667) | 14.97 (1.13) | SFV addiction | Multiple* | Loneliness, Well-being, Depression, Anxiety, Stress |
| Cheng 2023 [7] | Mixed-method | China | 420 (199) | NR | SFV addiction | NR | Anxiety, Negative mood |
| Chung 2025 [8] | Cross-sectional | China | 362 (197) | 18-34 | Daily use | NR | Occupational burnout, Well-being |
| Cui 2025 [9] | Cross-sectional | China | 684 (325) | 65.82 (7.05) | SFV addiction | NR | Loneliness, Neuroticism |
| Deng 2024 [10] | Cross-sectional | China | 264 couples | 68.02 (8.68) | SFV addiction | NR | Negative mood |
| Ding 2024 [11] | Cross-sectional | China | 694 (311) | 18—25 | SFV addiction, Daily use | NR | Well-being |
| Dong 2025 [12] | Cross-sectional | China | 872 (356) | Over 65 | SFV addiction | NR | Depression |
| Drivas 2024 [13] | Interventional | USA | 316 (116) | 19.80 (1.40) | Daily use | TikTok | Positive mood, Negative mood |
| Hong 2025 [14] | Cross-sectional | China | 2822 (779) | 20.39 (1.80) | SFV addiction | Multiple* | Stress |
| Hou 2024 [15] | Cross-sectional | China | 169 (61) | 21—30 | SFV addiction | TikTok | Anxiety, Loneliness |
| Hu 2024 [16] | Cross-sectional | China | 319 (148) | NR | Daily use | NR | Stress |
| Huang 2022 [17] | Cross-sectional | China | 202 (103) | 49.93 (3.05) | Problematic use, Daily use | NR | Stress |
| Jiang 2024 [18] | Cross-sectional | China | 1772 (787) | 15.30 (3.20) | SFV addiction | NR | Anxiety |
| Li 2023 [19] | Cross-sectional | China | 1203 (NR) | 15.41 (1.66) | SFV addiction | NR | Alexithymia |
| Li 2024a [20] | Cross-sectional | China | 931 (432) | 17.93 (0.79) | SFV addiction | NR | Depression |
| Li 2024b [21] | Cross-sectional | China | 1879 (689) | 19.46 (1.11) | SFV addiction | NR | Anxiety, Mindfulness |
| Li 2024c [22] | Cross-sectional | China | 8752 (4306) | 58.60 (9.25) | Daily use | NR | Depression |
| Liu 2021 [23] | Cross-sectional | China | 990 (473) | 19.76 (0.88) | SFV addiction | NR | Stress |

| **Table 1 Continued** | | | | | | | |
| --- | --- | --- | --- | --- | --- | --- | --- |
| **Study** | **Design** | **Country** | **Sample (n, male)** | **Age (SD)** | **SFV use** | **Platform** | **Mental health outcomes** |
| Liu 2024a [24] | Cross-sectional | China | 8929 (4537) | Over 45 | Daily use | NR | Depression |
| Liu 2024b [25] | Cross-sectional | China | 1263 (697) | 14.35 (1.66) | Daily use | NR | Negative mood, Well-being, Depression |
| Liu 2025 [26] | Prospective | China | 590 (303) | 19.83 (1.24) | Daily use | TikTok | Anxiety |
| Lu 2022 [27] | Cross-sectional | China | 96 (51) | 15-25 | SFV addiction | TikTok | Boredom |
| Mao 2025 [28] | Cross-sectional | China | 523 (200) | NR | SFV addiction | NR | Anxiety |
| Mu 2022 [29] | Cross-sectional | China | 242 (83) | Underage | SFV addiction | Multiple* | Well-being |
| Mu 2025 [30] | Cross-sectional | China | 986 (356) | 30.1 (7.7) | SFV addiction | NR | Emptiness |
| Peng 2025 [31] | Cross-sectional | China | 1002 (NR) | NR | SFV addiction | NR | Depression |
| Sun 2024 [32] | Cross-sectional | China | 422 (NR) | 26.55 (11.38） | SFV addiction | Multiple* | Stress |
| Tian 2023 [33] | Cross-sectional | China | 382 (172) | NR | SFV addiction | TikTok | Positive mood, Negative mood |
| Tu 2023 [34] | Cross-sectional | China | 596 (306) | 14.80 (1.85) | SFV addiction | NR | Stress |
| Wang 2023 [35] | Prospective | China | 858 (455) | 11.69 (1.11) | SFV addiction | NR | Emotional dysregulation |
| Wang 2024a [36] | Cross-sectional | China | 4213 (2212) | 16.41 (0.77) | SFV addiction | NR | Anxiety |
| Wang 2024b [37] | Cross-sectional | China | 524 (167) | 21.25 (3.49) | SFV addiction | TikTok | Loneliness |
| Wen 2024 [38] | Cross-sectional | China | 1209 (596) | 70.65 (6.40) | Perceived Overload | NR | Well-being, Loneliness |
| Wu 2021 [39] | Cross-sectional | China | 596 (335) | 19.64 (1.10) | Daily use | NR | Well-being, Positive mood |
| Wu 2025 [40] | Cross-sectional | China | 4750 (2952) | 16.01 (0.76) | SFV addiction | NR | Positive mood, Negative mood, Depression |
| Xia 2023 [41] | Cross-sectional | China | 1016 (NR) | NR | Daily use | NR | Depression, Anxiety |
| Xie 2023 [42] | Cross-sectional | China | 1047 (593) | 20.08 (1.34) | SFV addiction | NR | Boredom |
| Xu 2024 [43] | Prospective | China | 480 (250) | 14.76 (1.84) | SFV addiction | NR | Depression |
| Yang 2022 [44] | Cross-sectional | China | 490 (267) | NR | SFV addiction | NR | Emotion suppression |
| Yang 2025 [45] | Cross-sectional | China | 5785 (2822) | 15.75 (0.62) | SFV addiction | NR | Depression, Anxiety, Neuroticism |

| **Table 1 Continued** | | | | | | | |
| --- | --- | --- | --- | --- | --- | --- | --- |
| **Study** | **Design** | **Country** | **Sample (n, male)** | **Age (SD)** | **SFV use** | **Platform** | **Mental health outcomes** |
| Yu 2024 [46] | Cross-sectional | China | 1009 (522) | 19.80 (NR) | Problematic use | Multiple* | Suicidal ideation, Depression |
| Yue 2024 [47] | Cross-sectional | China | 336 (139) | 19.41 (0.94) | SFV addiction | NR | Loneliness |
| Zhai 2024 [48] | Cross-sectional | China | 1086 (465) | 20.95 (1.94) | Daily use | NR | Well-being, Positive mood, Negative mood, Anxiety |
| Zhang 2023 [49] | Cross-sectional | China | 1369 (582) | NR | SFV dependence | NR | Anxiety, Depression |
| Zhang 2024a [50] | Prospective | China | 331 (185) | NR | Daily use | NR | Depression |
| Zhang 2024b [51] | Cross-sectional | China | 3062 (1623) | 68.32 (5.88) | Daily use | Multiple* | Depression |
| Zhang 2024c [52] | Cross-sectional | China | 1111 (NR) | 20.4 (1.70) | Overuse | NR | Depression |
| Zhang 2024d [53] | Cross-sectional | China | 532 (207) | 19.32 (1.23) | SFV addiction | NR | Boredom |
| Zhao 2024 [54] | Cross-sectional | China | 388 (121) | 19.95 (1.02) | SFV addiction | NR | Loneliness |
| Zhou 2024a [55] | Cross-sectional | China | 4787 (2478) | 68.17 (5.82) | Daily use | NR | Depression, Well-being |
| Zhou 2024b [56] | Cross-sectional | China | 632 (330) | 21.94 (2.64) | Problematic use | Multiple* | Boredom |
| Zhu 2024 [57] | Cross-sectional | China | 1302 (555) | 16.03 (0.76) | SFV addiction, Daily use | NR | Depression |
| Zuo 2024 [58] | Cross-sectional | China | 16765 (8517) | 47.86 (18—95) | Daily use | NR | Well-being |

NR: Not reported; SFV: Short-form videos.

* Indicates that the study was conducted across multiple SFV platforms (including TikTok, Instagram, YouTube, Sina Weibo, Xiaohongshu, Xigua Video, Pear Video, Kuaishou, Watermelon Video, Huoshan Video, Tencent Wesee).

**Reference list (Included studies)**

1 Abulibdeh ES, Alneyadi S, Skaik H, *et al.* Short VideoAddiction and Its Relationship with Students’ Academic Achievement and Well-Being: A Pilot Study. *2024 Global Digital Health Knowledge Exchange & Empowerment Conference (gDigiHealth.KEE)*. 2024:1–6.

2 Al Azri M, Al Harrasi A, Al Ghailani A, *et al.* The moderating roles of social comparison in the relationship between Instagram reels use and mental health outcomes: a cross-sectional study in Oman. *Sci Rep*. 2025;15:39457. doi: 10.1038/s41598-025-22938-8

3 Asad K, Ali F, Awais M. Personality Traits, Narcissism and TikTok Addiction: A Parallel Mediation Approach. *International Journal of Media and Information Literacy*. 2022;7:293–304. doi: 10.13187/ijmil.2022.2.293

4 Bai J, Mo K, Peng Y, *et al.* The Relationship Between the Use of Mobile Social Media and Subjective Well-Being: The Mediating Effect of Boredom Proneness. *Frontiers in Psychology*. 2021;11. doi: 10.3389/fpsyg.2020.568492

5 Baltacı Ö, Açar I. Does Leisure Boredom Predict Short Video Addiction in Adolescents? *Psychiatr Q*. Published Online First: 12 June 2025. doi: 10.1007/s11126-025-10172-4

6 Chao M, Lei J, He R, *et al.* TikTok use and psychosocial factors among adolescents: Comparisons of non-users, moderate users, and addictive users. *Psychiatry Research*. 2023;325:115247. doi: 10.1016/j.psychres.2023.115247

7 Cheng X, Su X, Yang B, *et al.* Understanding users’ negative emotions and continuous usage intention in short video platforms. *Electronic Commerce Research and Applications*. 2023;58:101244. doi: 10.1016/j.elerap.2023.101244

8 Chung D, Meng Y, Wang J. The Role of Short-Form Video Apps in Mitigating Occupational Burnout and Enhancing Life Satisfaction Among Healthcare Workers: A Serial Multiple Mediation Model. *Healthcare (Basel)*. 2025;13:355. doi: 10.3390/healthcare13040355

9 Cui S, Jiang J, Mu L. Impact Mechanism of Adult Offspring’s Phubbing Behavior on Elderly Short Video Addiction: A Moderated Mediation Model. *Applied Research Quality Life*. Published Online First: 8 January 2025. doi: 10.1007/s11482-024-10405-x

10 Deng J, Wang M, Mu W, *et al.* The Relationship between Addictive Use of Short-Video Platforms and Marital Satisfaction in Older Chinese Couples: An Asymmetrical Dyadic Process. *Behavioral Sciences*. 2024;14:364. doi: 10.3390/bs14050364

11 Ding J, Hu Z, Zuo Y, *et al.* The relationships between short video addiction, subjective well-being, social support, personality, and core self-evaluation: a latent profile analysis. *BMC Public Health*. 2024;24:3459. doi: 10.1186/s12889-024-20994-9

12 Dong P, Zhang X, Yin W, *et al.* Relationship Between Short Video Addiction Tendency and Depression Among Rural Older Adults: Cross-Sectional Study. *Journal of Medical Internet Research*. 2025;27:e75938. doi: 10.2196/75938

13 Drivas M, Reed OS, Berndt-Goke M. #WhatIEatInADay: The effects of viewing food diary TikTok videos on young adults’ body image and intent to diet. *Body Image*. 2024;49:101712. doi: 10.1016/j.bodyim.2024.101712

14 Hong X, Du W, Quan X, *et al.* Network analysis of the interactions of short video features and personal characteristics for short video addiction in Chinese young adults. *Acta Psychologica*. 2025;260:105482. doi: 10.1016/j.actpsy.2025.105482

15 Hou A, Hou X. Enjoyment or loneliness: The influence of cognitive absorption on addiction to short-form video apps. *Bulletin of Educational Psychology*. 2024;55:537–56. doi: 10.6251/BEP.202403_55(3).0005

16 Hu H, Huang M. How stress influences short video addiction in China: an extended compensatory internet use model. *Front Psychol*. 2024;15. doi: 10.3389/fpsyg.2024.1470111

17 Huang Q, Hu M, Chen H. Exploring Stress and Problematic Use of Short-Form Video Applications among Middle-Aged Chinese Adults: The Mediating Roles of Duration of Use and Flow Experience. *International Journal of Environmental Research and Public Health*. 2022;19:132. doi: 10.3390/ijerph19010132

18 Jiang Y, Feng Y, Qi J, *et al.* The relationship between bullying victimization and academic performance among adolescents: The chain mediating roles of social anxiety and short video addiction. *Psychology in the Schools*. 2024;61:3859–72. doi: 10.1002/pits.23267

19 Li F, Wang G, Zhu D, *et al.* Parental neglect and short-form video application addiction in Chinese adolescents: The mediating role of alexithymia and the moderating role of refusal self-efficacy. *Child Abuse & Neglect*. 2023;143:106345. doi: 10.1016/j.chiabu.2023.106345

20 Li L, Li X, Li Y, *et al.* Types of short video addiction among college freshmen: Effects on career adaptability, insomnia, and depressive symptoms. *Acta Psychologica*. 2024;248:104380. doi: 10.1016/j.actpsy.2024.104380

21 Li G, Geng Y, Wu T. Effects of short-form video app addiction on academic anxiety and academic engagement: The mediating role of mindfulness. *Front Psychol*. 2024;15. doi: 10.3389/fpsyg.2024.1428813

22 Li C, and Wang Y. Short-Form Video Applications Usage and Functionally Dependent Adults’ Depressive Symptoms: A Cross-Sectional Study Based on a National Survey. *Risk Management and Healthcare Policy*. 2024;17:3099–111. doi: 10.2147/RMHP.S491498

23 Liu Y, Ni X, Niu G. Perceived Stress and Short-Form Video Application Addiction: A Moderated Mediation Model. *Front Psychol*. 2021;12:747656. doi: 10.3389/fpsyg.2021.747656

24 Liu L, Wang Y. The influence of watching short videos on the mental health of the middle-aged and elderly in China. *Modern Preventive Medicine*. 2024;51:111–7. doi: 10.20043/j.cnki.MPM.202305090

25 Liu M, Zhuang A, Norvilitis JM, *et al.* Usage patterns of short videos and social media among adolescents and psychological health: A latent profile analysis. *Computers in Human Behavior*. 2024;151:108007. doi: 10.1016/j.chb.2023.108007

26 Liu Q, Wen J, Wang N, *et al.* Longitudinal Associations Between TikTok Use, Self-Concept Clarity, and Anxiety Among Chinese Emerging Adults: Exploring Differential Impacts of Active and Passive TikTok Use. *Emerging Adulthood*. 2025;13:265–77. doi: 10.1177/21676968241305653

27 Lu L, Liu M, Ge B, *et al.* Adolescent Addiction to Short Video Applications in the Mobile Internet Era. *Front Psychol*. 2022;13. doi: 10.3389/fpsyg.2022.893599

28 Mao M, Liao F. Undergraduates short form video addiction and learning burnout association involving anxiety symptoms and coping styles moderation. *Sci Rep*. 2025;15:24191. doi: 10.1038/s41598-025-09656-x

29 Mu H, Jiang Q, Xu J, *et al.* Drivers and Consequences of Short-Form Video (SFV) Addiction amongst Adolescents in China: Stress-Coping Theory Perspective. *International Journal of Environmental Research and Public Health*. 2022;19:14173. doi: 10.3390/ijerph192114173

30 Mu W, Kong L, He A. Understanding Emptiness in a Chinese Sample: Cross-cultural Validation, Latent Profile Analysis, and Association with Short-Form Video Addiction. *Int J Ment Health Addiction*. 2025;23:3895–916. doi: 10.1007/s11469-024-01327-8

31 Peng B, Zhu X, Han T, *et al.* Stepping away from the scroll: a chain mediation model from physical exercise and adolescent short video addiction. *BMC Psychol*. 2025;13:1366. doi: 10.1186/s40359-025-03710-z

32 Sun R, Zhang MX, Yeh C, *et al.* The metacognitive-motivational links between stress and short-form video addiction. *Technology in Society*. 2024;77:102548. doi: 10.1016/j.techsoc.2024.102548

33 Tian X, Bi X, Chen H. How short-form video features influence addiction behavior? Empirical research from the opponent process theory perspective. *Information Technology & People*. 2022;36:387–408. doi: 10.1108/ITP-04-2020-0186

34 Tu W, Nie Y, Liu Q, *et al.* Does the Effect of Stress on Smartphone Addiction Vary Depending on the Gender and Type of Addiction? *Behavioral Sciences*. 2023;13. doi: 10.3390/bs13100810

35 Wang J, Wang M, Lei L. Longitudinal links among paternal and maternal harsh parenting, adolescent emotional dysregulation and short-form video addiction. *Child Abuse & Neglect*. 2023;141:106236. doi: 10.1016/j.chiabu.2023.106236

36 Wang P, Ouyang M, Yin L, *et al.* Gender differences in the relationship between parent-adolescent attachment anxiety and adolescent short-form video addiction: the mediating role of fear of missing out. *Curr Psychol*. 2024;43:24216–28. doi: 10.1007/s12144-024-06060-y

37 Wang P, Yue Y, Ouyang M, *et al.* Do Narcissistic People Exhibit More Authentic Self-Disclosure to Generative AI? The Roles of Short-Form Video Addiction, Loneliness, and Usage Intention. *Social Science Computer Review*. 2024;08944393241308511. doi: 10.1177/08944393241308511

38 Wen X, Zhou ,Yaping, Li ,Yinan, *et al.* Perceived Overload on Short Video Platforms and Its Influence on Mental Health Among the Elderly: A Moderated Mediation Model. *Psychology Research and Behavior Management*. 2024;17:2347–62. doi: 10.2147/PRBM.S459426

39 Wu Y, Wang X, Hong S, *et al.* The relationship between social short-form videos and youth’s well-being: It depends on usage types and content categories. *Psychology of Popular Media*. 2021;10:467–77. doi: 10.1037/ppm0000292

40 Wu Y, Bai Y, Liu X, *et al.* Gender Differences in the Relationship Between Short-Form Video Addiction and Adolescent Depression: The Mediating Role of Attentional Bias. *Cyberpsychology, Behavior, and Social Networking*. 2025;28:169–77. doi: 10.1089/cyber.2024.0442

41 Xia L-L, Li J-D, Duan F, *et al.* Effects of Online Game and Short Video Behavior on Academic Delay of Gratification - Mediating Effects of Anxiety, Depression and Retrospective Memory. *Psychology Research and Behavior Management*. 2023;16:4353–65. doi: 10.2147/PRBM.S432196

42 Xie J, Xu X, Zhang Y, *et al.* The effect of short-form video addiction on undergraduates’ academic procrastination: a moderated mediation model. *Front Psychol*. 2023;14. doi: 10.3389/fpsyg.2023.1298361

43 Xu X, Li H, Bai R, *et al.* Do Boys and Girls Display Different Levels of Depression in Response to Mobile Phone Addiction? Examining the Longitudinal Effects of Four Types of Mobile Phone Addiction. *Psychology Research and Behavior Management*. 2024;17:4315–29. doi: 10.2147/PRBM.S487298

44 Yang J, Ti Y, Ye Y. Offline and Online Social Support and Short-Form Video Addiction Among Chinese Adolescents: The Mediating Role of Emotion Suppression and Relatedness Needs. *Cyberpsychology, Behavior, and Social Networking*. 2022;25:316–22. doi: 10.1089/cyber.2021.0323

45 Yang C, Du J, Li X, *et al.* Association between parental phubbing and short-form video addiction: A moderated mediation analysis among Chinese adolescents. *Journal of Affective Disorders*. 2025;369:523–30. doi: 10.1016/j.jad.2024.10.023

46 Yu Z, Zhu X, Li Y. The association between problematic short video use and suicidal ideation and self-injurious behaviors: the mediating roles of sleep disturbance and depression. *BMC Public Health*. 2024;24:1689. doi: 10.1186/s12889-024-19191-5

47 Yue H, Yang G, Bao H, *et al.* Linking negative cognitive bias to short-form video addiction: The mediating roles of social support and loneliness. *Psychology in the Schools*. 2024;61:4026–40. doi: 10.1002/pits.23260

48 Zhai G, Su J, Chen Z, *et al.* The Relationships Between Short Video Usage and Subjective Well-Being: Mediation Models and Network Analysis. *Behavioral Sciences*. 2024;14:1082. doi: 10.3390/bs14111082

49 Zhang Q, Wang A, Zhang J. Relationship between social short-form video usage and mental health-related behaviors among college students. *Chin J Sch Health*. 2023;44:586–9. doi: 10.16835/j.cnki.1000-9817.2023.04.025

50 Zhang D, Yang Y, Guan M. A cross-lagged analysis of the relationship between short video overuse behavior and depression among college students. *Front Psychol*. 2024;15:1345076. doi: 10.3389/fpsyg.2024.1345076

51 Zhang R, Su Y, Lin Z, *et al.* The impact of short video usage on the mental health of elderly people. *BMC Psychol*. 2024;12:612. doi: 10.1186/s40359-024-02125-6

52 Zhang D, Wu J, Li J, *et al.* Influence of short video overuse behavior on depression of college students: the chain mediating effect of self-concept clarity and meaning in life. *Chinese Journal of Behavioral Medicine and Brain Science*. 2024;33:342–6. doi: 10.3760/cma.j.cn371468-20230921-00125

53 Zhang Y, Bu ,Ruohan, and Li X. Social Exclusion and Short Video Addiction: The Mediating Role of Boredom and Self-Control. *Psychology Research and Behavior Management*. 2024;17:2195–203. doi: 10.2147/PRBM.S463240

54 Zhao Z, Kou Y. Effects of loneliness on short video addiction among college students: the chain mediating role of social support and physical activity. *Front Public Health*. 2024;12:1484117. doi: 10.3389/fpubh.2024.1484117

55 Zhou D, Li W. Never Say too Late: How Older Adults’ Short Video Watching Behavior Affects Their Mental Health? *Documentation, Information and Knowledge*. 2024;41:36–45 and 64. doi: 10.13366/j.dik.2024.05.036

56 Zhou L, Lv X, Zhou Y, *et al.* A Network Analysis Perspective on the Relationship Between Boredom, Attention Control, and Problematic Short Video Use Among a Sample of Chinese Young Adults. *Int J Ment Health Addiction*. Published Online First: 18 September 2024. doi: 10.1007/s11469-024-01392-z

57 Zhu C, Jiang Y, Lei H, *et al.* The relationship between short-form video use and depression among Chinese adolescents: Examining the mediating roles of need gratification and short-form video addiction. *Heliyon*. 2024;10. doi: 10.1016/j.heliyon.2024.e30346

58 Zuo X, Wang R, Hong Z. Does using short video apps impacts life satisfaction: a perspective from psycho-social mechanism. *Curr Psychol*. 2024;43:21242–56. doi: 10.1007/s12144-024-05896-8
