# Supplementary material for: Association Between Short-Form Video Use and Mental Health: Systematic Review and Meta-Analysis
Source: J Med Internet Res. 2026 Mar 18;28:e82503. doi: 10.2196/82503 (PMC12998613; doi:10.2196/82503)
Supplement: Multimedia Appendix 3 [file jmir-v28-e82503-s003.docx]

**Supplemental 3 Quality assessment of included studies**

**Joanna Briggs Quality Assessment Items**

1. Were the criteria for inclusion in the sample clearly defined?
2. Were the study subjects and the setting described in detail?
3. Was the exposure measured in a valid and reliable way?
4. Were objective, standard criteria used for measurement of the condition?
5. Were confounding factors identified?
6. Were strategies to deal with confounding factors stated?
7. Were the outcomes measured in a valid and reliable way?
8. Was appropriate statistical analysis used?

N: negative answer (no), Y: positive answer (yes), U: unclear/partial (unclear)

| **Study** | **1** | **2** | **3** | **4** | **5** | **6** | **7** | **8** | **Total**  **(Y%)** |
| --- | --- | --- | --- | --- | --- | --- | --- | --- | --- |
| Abulibdeh 2024 | N | Y | Y | Y | N | N | Y | U | 50% |
| Al Azri 2025 | Y | Y | Y | Y | U | Y | Y | Y | 87.5% |
| Asad 2022 | Y | N | Y | Y | N | N | Y | Y | 62.5% |
| Bai 2021 | U | Y | Y | Y | Y | Y | Y | Y | 87.5% |
| Baltacı 2025 | Y | Y | Y | Y | N | N | Y | Y | 75% |
| Chao 2023 | U | Y | Y | Y | Y | Y | Y | Y | 87.5% |
| Cheng 2023 | N | Y | N | N | Y | Y | Y | Y | 62.5% |
| Chung 2025 | Y | Y | Y | N | Y | Y | Y | Y | 87.5% |
| Cui 2025 | Y | Y | N | Y | Y | Y | Y | Y | 87.5% |
| Deng 2024 | Y | Y | Y | Y | Y | Y | Y | Y | 100% |
| Ding 2024 | Y | Y | Y | Y | N | N | Y | Y | 75% |
| Dong 2025 | Y | Y | Y | Y | Y | Y | Y | Y | 100% |
| Drivas 2024 | U | Y | N | N | Y | Y | Y | Y | 62.5% |
| Hong 2025 | U | Y | Y | Y | N | N | Y | Y | 62.5% |
| Hou 2024 | Y | Y | Y | Y | N | N | Y | U | 62.5% |
| Hu 2024 | Y | Y | N | Y | N | N | Y | Y | 62.5% |
| Huang 2022 | Y | Y | N | Y | Y | Y | Y | Y | 87.5% |
| Jiang 2024 | U | Y | Y | Y | Y | Y | U | Y | 75% |
| Li 2023 | Y | Y | Y | Y | Y | Y | Y | Y | 100% |
| Li 2024a | Y | Y | Y | Y | Y | Y | Y | Y | 100% |
| Li 2024b | N | Y | Y | Y | Y | Y | Y | Y | 87.5% |
| Li 2024c | Y | Y | N | N | Y | Y | U | Y | 62.5% |
| Liu 2021 | Y | Y | N | Y | N | N | Y | Y | 62.5% |
| Liu 2024a | U | Y | N | N | Y | Y | Y | Y | 62.5% |
| Liu 2024b | U | Y | Y | Y | Y | Y | Y | Y | 87.5% |
| Liu 2025 | Y | Y | Y | Y | Y | Y | Y | Y | 100% |
| Lu 2022 | Y | Y | Y | Y | Y | Y | Y | U | 87.5% |
| Mao 2025 | Y | Y | Y | Y | N | N | Y | Y | 75% |
| Mu 2022 | Y | Y | Y | Y | Y | Y | Y | Y | 100% |
| Mu 2025 | Y | Y | Y | Y | N | N | Y | Y | 75% |
| Peng 2025 | Y | Y | Y | Y | Y | U | Y | Y | 87.5% |
| Sun 2024 | Y | Y | Y | Y | Y | Y | Y | Y | 100% |
| Tian 2023 | Y | Y | Y | Y | Y | Y | Y | Y | 100% |
| Tu 2023 | Y | Y | Y | Y | Y | Y | Y | Y | 100% |
| Wang 2023 | N | Y | Y | Y | N | N | Y | Y | 62.5% |
| Wang 2024a | N | Y | Y | Y | Y | Y | Y | Y | 87.5% |
| Wang 2024b | Y | Y | N | N | Y | Y | Y | Y | 75% |
| Wen 2024 | Y | Y | N | N | Y | Y | Y | Y | 75% |
| Wu 2021 | U | Y | N | N | Y | Y | Y | Y | 62.5% |
| Wu 2025 | U | Y | Y | Y | Y | Y | Y | Y | 87.5% |
| Xia 2023 | Y | Y | Y | Y | U | U | Y | Y | 75% |
| Xie 2023 | N | Y | Y | Y | Y | Y | Y | Y | 87.5% |
| Xu 2024 | Y | Y | Y | Y | Y | U | Y | Y | 87.5% |
| Yang 2022 | N | Y | Y | Y | N | N | Y | Y | 62.5% |
| Yang 2025 | Y | Y | Y | Y | Y | Y | Y | Y | 100% |
| Yu 2024 | Y | Y | Y | Y | N | N | Y | Y | 75% |
| Yue 2024 | N | Y | Y | Y | N | N | Y | Y | 62.5% |
| Zhai 2024 | N | Y | Y | Y | N | N | Y | Y | 62.5% |
| Zhang 2023 | N | Y | Y | Y | N | N | Y | Y | 62.5% |
| Zhang 2024a | N | N | Y | Y | Y | Y | Y | Y | 75% |
| Zhang 2024b | Y | Y | N | N | Y | Y | Y | Y | 75% |
| Zhang 2024c | U | Y | Y | Y | Y | Y | Y | Y | 87.5% |
| Zhang 2024d | N | Y | Y | Y | N | N | Y | Y | 62.5% |
| Zhao 2024 | Y | Y | Y | Y | Y | Y | Y | Y | 100% |
| Zhou 2024a | Y | Y | N | N | Y | Y | Y | Y | 75% |
| Zhou 2024b | Y | Y | Y | Y | N | N | Y | Y | 75% |
| Zhu 2024 | Y | Y | N | Y | Y | Y | Y | Y | 87.5% |
| Zuo 2024 | N | Y | N | N | Y | Y | Y | Y | 62.5% |
| Total (Y%) | 60.3% | 96.6% | 74.1% | 81% | 65.5% | 63.7% | 96.6% | 94.8% | 79.1% |
