# Supplementary material for: Association Between Short-Form Video Use and Mental Health: Systematic Review and Meta-Analysis
Source: J Med Internet Res. 2026 Mar 18;28:e82503. doi: 10.2196/82503 (PMC12998613; doi:10.2196/82503)
Supplement: Multimedia Appendix 4 [file jmir-v28-e82503-s004.docx]

**Supplemental 4 Funnel plots**

**Subjective well-being**

**
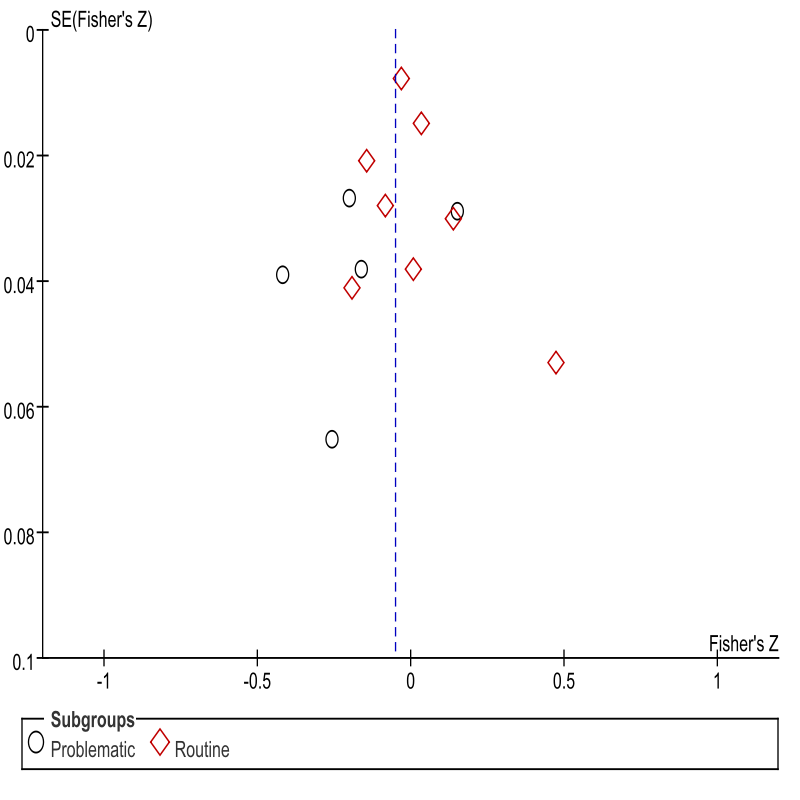
**

**Positive affect**

**
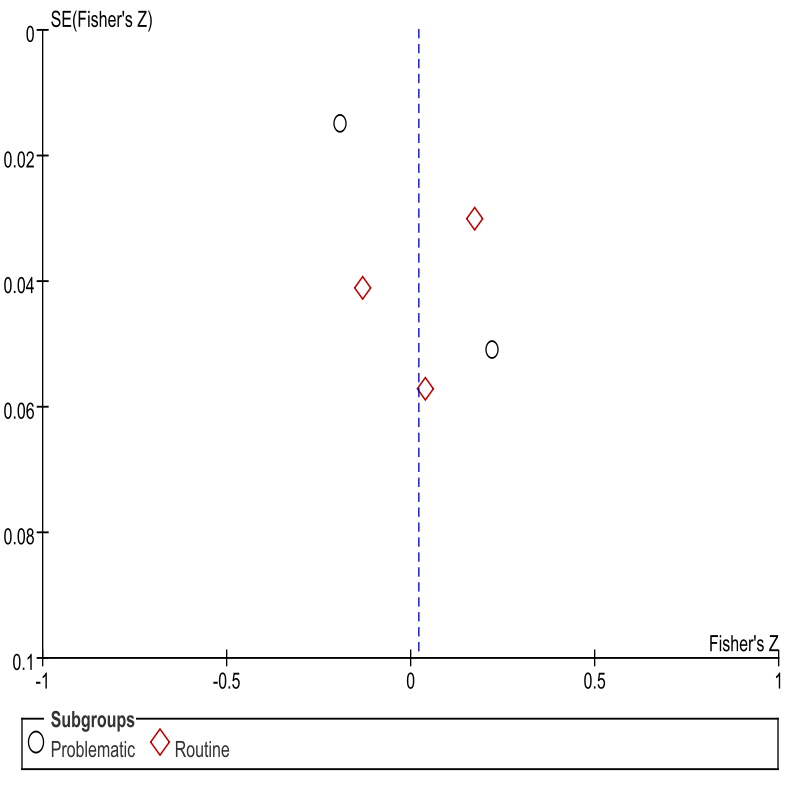
**

**Depression**

**
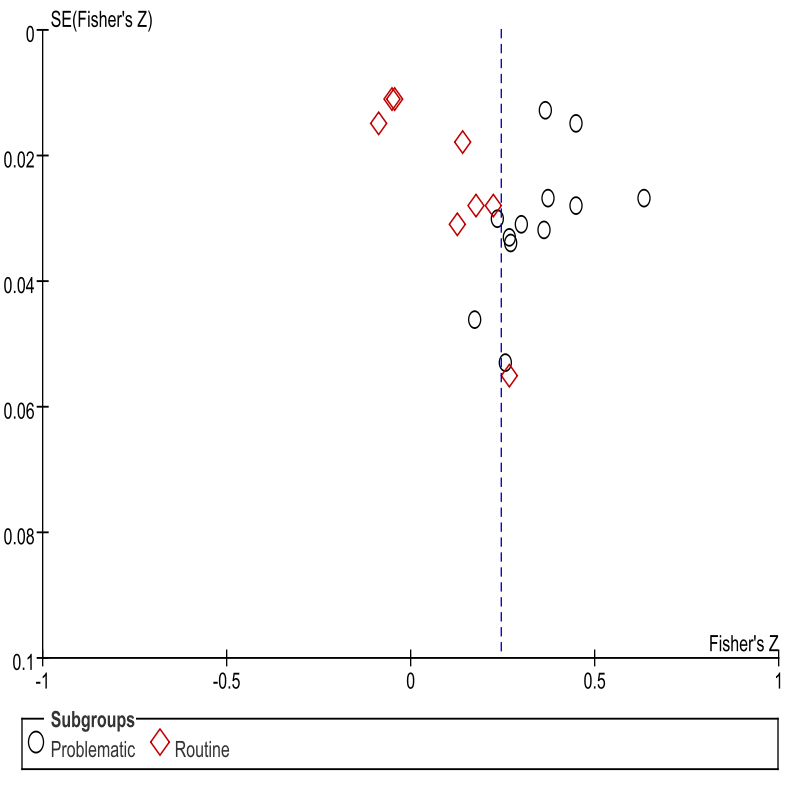
**

**Anxiety**

**
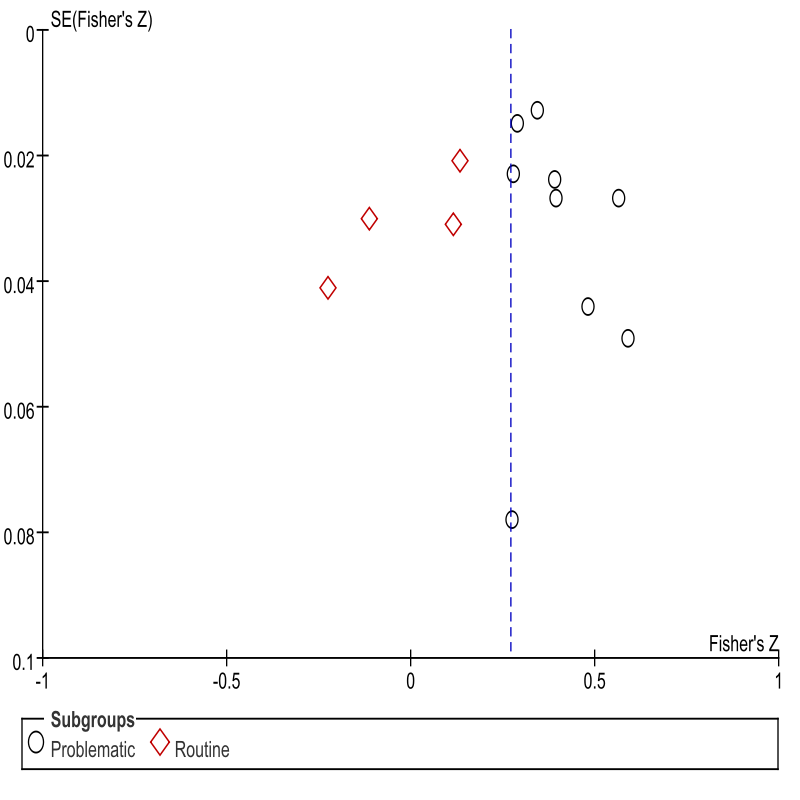
**

**Stress**

**
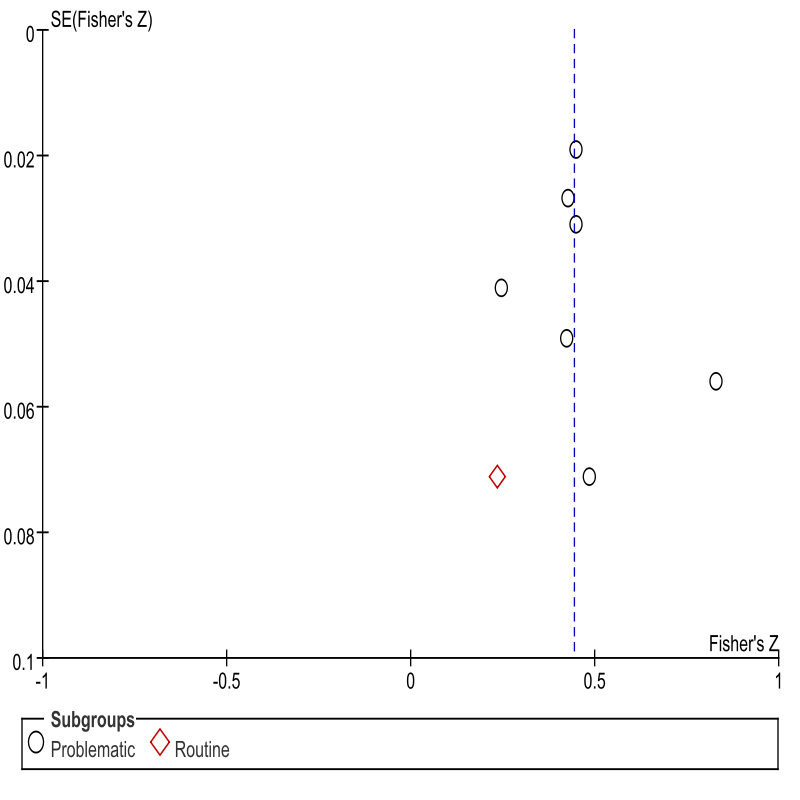
**

**Negative affect**

**
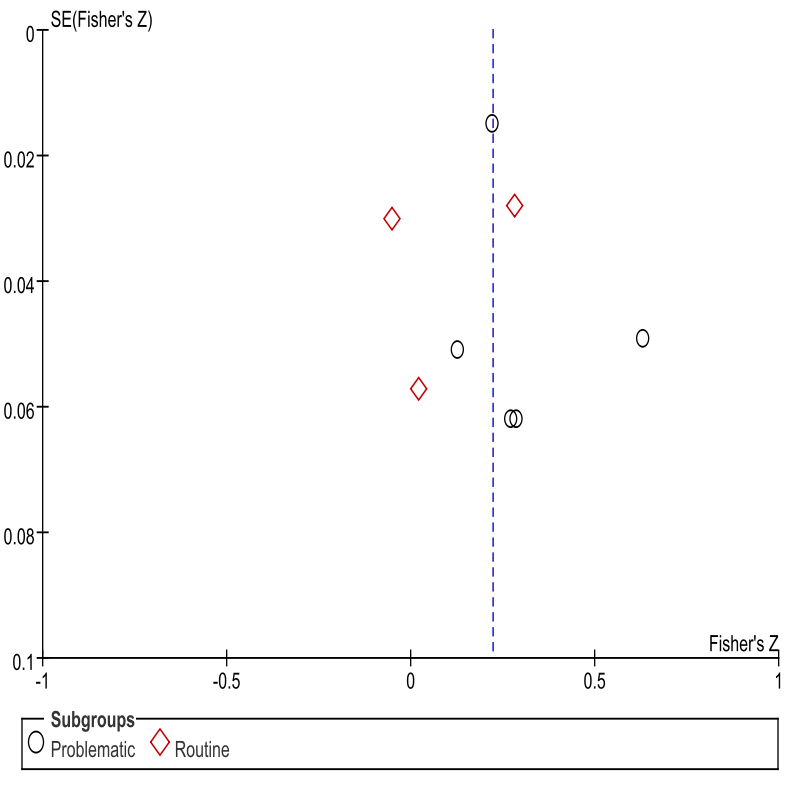
**

**Loneliness**

**
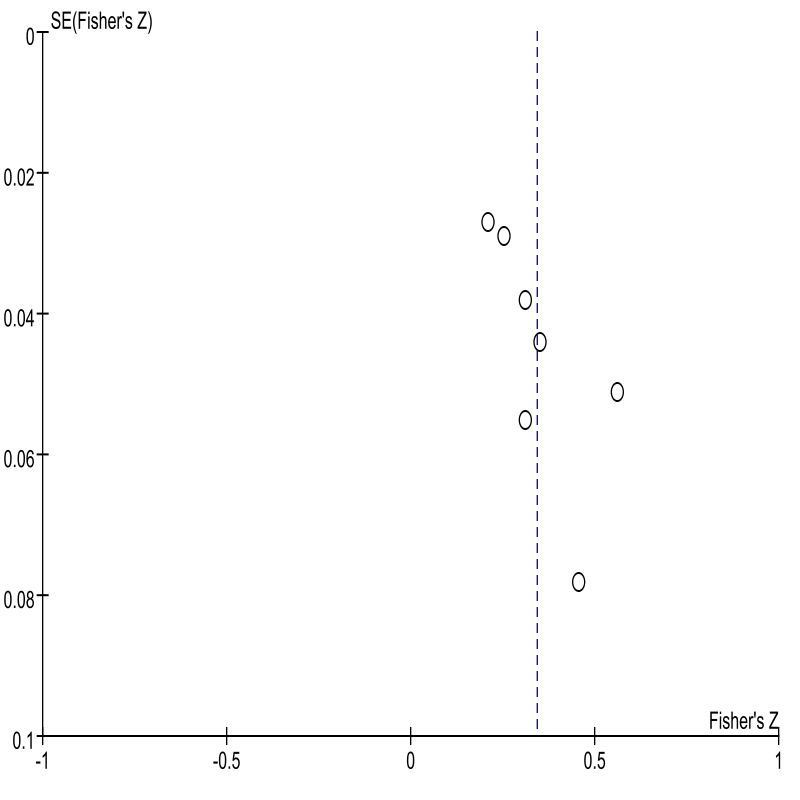
**

**Boredom**

**
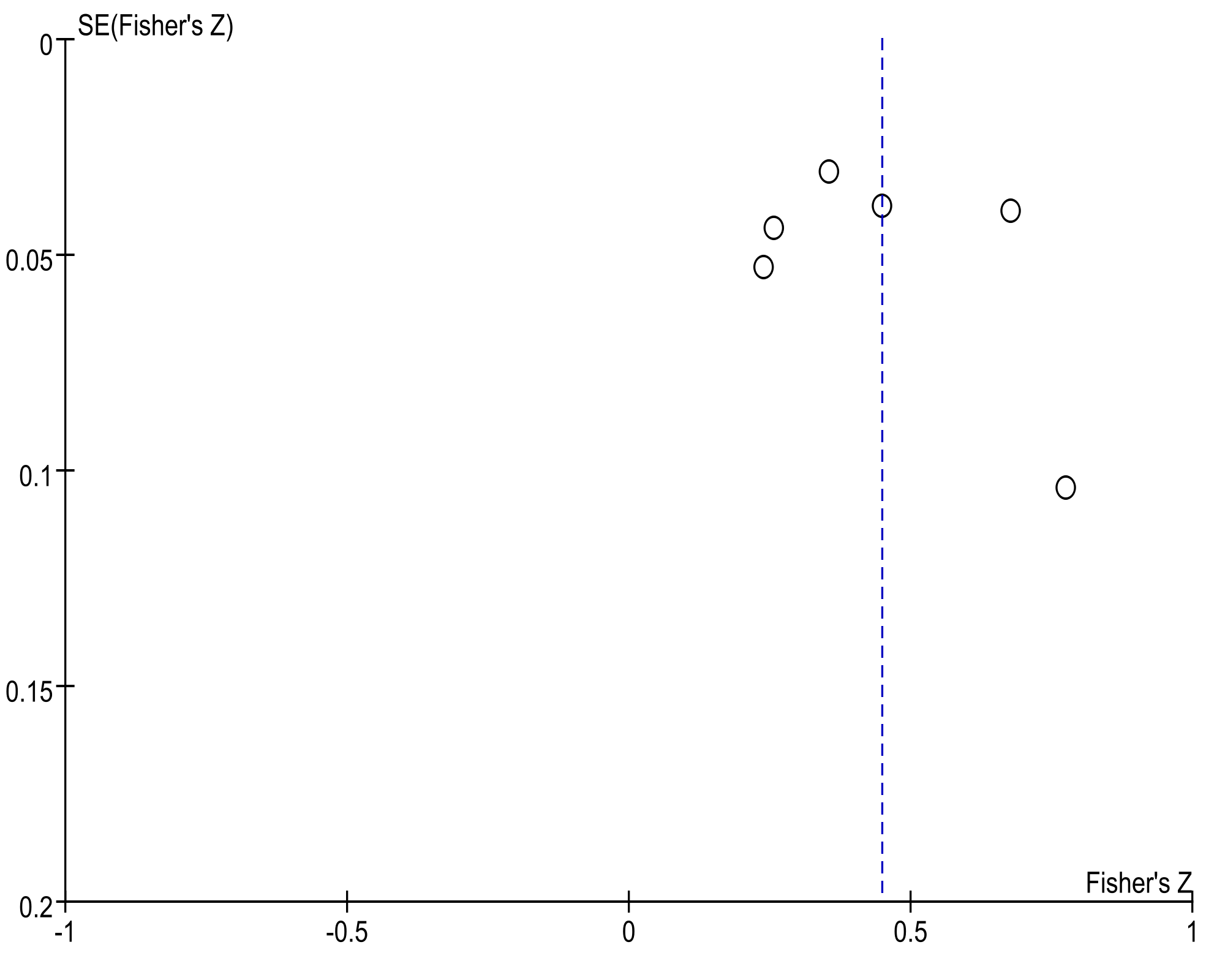
**
